# CIDP: a multi-functional platform for designing CRISPR sgRNAs

**DOI:** 10.1093/hr/uhad092

**Published:** 2023-05-04

**Authors:** Dong Xu, Jin Zhang, Xianjia Zhao, Yuze Hou, Heling Jiang, Wenchuang He, Xiongfeng Ma, Weihua Pan

**Affiliations:** Shenzhen Branch, Guangdong Laboratory for Lingnan Modern Agriculture, Genome Analysis Laboratory of the Ministry of Agriculture and Rural Affairs, Agricultural Genomics Institute at Shenzhen, Chinese Academy of Agricultural Sciences, Shenzhen, 518120, China; State Key Laboratory of Subtropical Silviculture, College of Forestry and Biotechnology, Zhejiang A&F University, Hangzhou, Zhejiang 311300, China; Shenzhen Branch, Guangdong Laboratory for Lingnan Modern Agriculture, Genome Analysis Laboratory of the Ministry of Agriculture and Rural Affairs, Agricultural Genomics Institute at Shenzhen, Chinese Academy of Agricultural Sciences, Shenzhen, 518120, China; College of Data Science, Taiyuan University of Technology, Taiyuan, Shanxi 030600, China; Shenzhen Branch, Guangdong Laboratory for Lingnan Modern Agriculture, Genome Analysis Laboratory of the Ministry of Agriculture and Rural Affairs, Agricultural Genomics Institute at Shenzhen, Chinese Academy of Agricultural Sciences, Shenzhen, 518120, China; Shenzhen Branch, Guangdong Laboratory for Lingnan Modern Agriculture, Genome Analysis Laboratory of the Ministry of Agriculture and Rural Affairs, Agricultural Genomics Institute at Shenzhen, Chinese Academy of Agricultural Sciences, Shenzhen, 518120, China; State Key Laboratory of Cotton Biology, Institute of Cotton Research, Chinese Academy of Agricultural Sciences, Anyang 455000, China; Shenzhen Branch, Guangdong Laboratory for Lingnan Modern Agriculture, Genome Analysis Laboratory of the Ministry of Agriculture and Rural Affairs, Agricultural Genomics Institute at Shenzhen, Chinese Academy of Agricultural Sciences, Shenzhen, 518120, China

Dear Editor,

Compared with traditional technologies affecting gene expression, changing DNA sequences of target genes is one of the most outstanding characters of CRISPR (Clustered Regularly Interspaced Short Palindromic Repeats). Single-guide RNAs (sgRNAs) guiding endonuclease Cas to target sites is a crucial step of CRISPR-Cas system for changing DNA sequences. An ideal sgRNA should only bind to the target gene. However, similar sequences of non-target sites can also be recognized leading to off-target effects [1]. How to design sgRNAs to minimize off-target effects is a great challenge. Most sgRNA-design tools thus require users to select a reference species for evaluating off-target effects [2]. However, there are significant differences in sequences between species, even though they are closely related species. Meanwhile, the calculation algorithms of off-target scores are highly sensitive to the sequences of target and non-target sites [3]. Therefore, the off-target scores may not be correctly calculated, unless the reference is the studied species, whereas large amounts of studied species cannot be found in design tools; for example, the number of species contained in BE-Designer is 142 that is more than those of other popular tools (Table S1, see online supplementary material). However, to date, at least 1096 plant species genomes have been released [4], meaning that it is difficult to design sgRNAs for six in seven studied species. Instead of selecting a reference, here, we describe CRISPR Integrated Design Platform (CIDP), a novel software that allows users to build background datasets using genomic sequences for designing sgRNAs.

## New features

Compared with previous tools (Table S1, see online supplementary material), the main improvements added in CIDP are as follows. (i) The CIDP database for designing sgRNAs is generated using genomic sequences. In other words, CIDP can be used for any species with known genomic sequences. (ii) CIDP can search shared sgRNAs of a group of genes simultaneously, which may play important roles in multiplex editing for polygenic traits [5]. (iii) CIDP preferentially searches sgRNAs with sequence uniqueness across genome. Furthermore, the potential off-target sites with base differences less than five (user-settable) can be automatically detected, and off-target scores are thus calculated by cutting frequency determination algorithm [3]. (iv) CIDP was specially developed for wet-lab biologists. Given that some researchers may lack sufficient experience to extract sequences from a genome, we set extraction functions in CIDP to help users obtain the related sequences of target genes from genomic sequences, including promoters, mRNA, untranslated region (UTR), and so on. (v) CIDP can be utilized for designing sgRNAs in batch.

**Figure 1:**
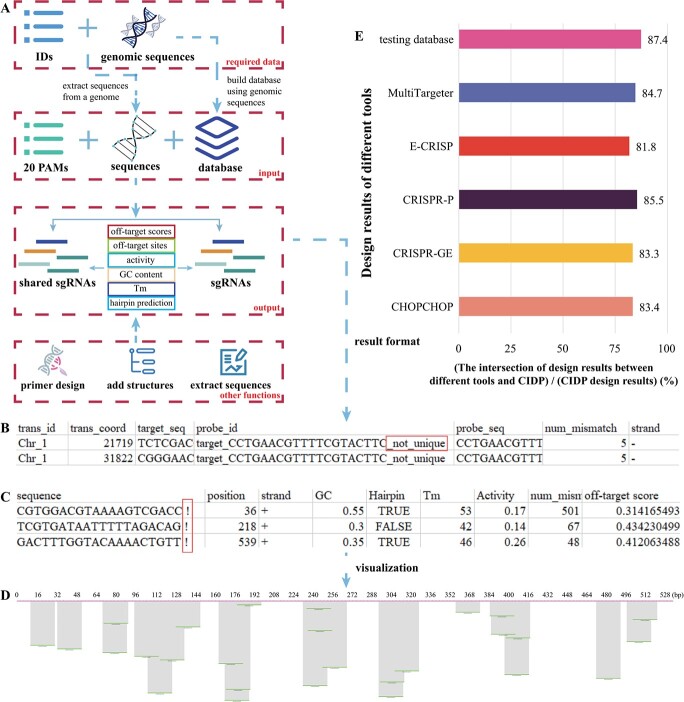
Designing sgRNAs using CRISPR Integrated Design Platform (CIDP). **A** The flow chart of CIDP. **B** and **C** show the alignment and sgRNA-design results of CIDP, respectively. **D** An example of CIDP visualization function. **E** Comparison of design results between CIDP and other tools.

## The workflow of CIDP

Fig. 1A shows the basic operations about using CIDP. Before designing sgRNAs, users need to build the sgRNA database containing all sgRNAs with unique sequences across the genome. CIDP allows users to directly input gene sequences for searching sgRNAs based on the chosen protospacer adjacent motif (PAM) model. We integrated 20 different PAM models [6] in CIDP for choice. After that, the candidate sgRNAs will be mapped into the sgRNA database to identify whether these sgRNAs have unique sequences. Furthermore, the obtained sgRNAs will be aligned into the genome to search potential off-target sites. Meanwhile, the parameters will be calculated to evaluate the efficiency, specificity, and activity of a sgRNA, such as hairpin structure [7], predicted activity [8], and off-target scores. In addition to designing sgRNAs, other functions of CIDP can be used for designing primers or adding special structures (including hairpin [7] and GOLD structure [9]) into sgRNAs automatically.

We set the visualization function to display the positions of sgRNAs on target genes (Fig. 1D). One output file of CIDP contains the information of potential off-target sites (such as their sequences and mismatch number, Fig. 1B). The ‘not_unique’ means that the sequence of this sgRNA is not unique in the genome. Another output file of CIDP shows sgRNAs and the relevant parameters (Fig. 1C). Especially, the related sgRNAs will be marked with an exclamatory mark if four consecutive ‘T’ or ‘A’s are detected in sequences.

## Evaluation of design results

In order to evaluate design results of CIDP, we randomly selected 1000 genes of *Arabidopsis*, and then used CHOPCHOP (http://chopchop.cbu.uib.no/), CRISPR-GE (http://skl.scau.edu.cn/targetdesign/), CRISPR-P (http://crispr.hzau.edu.cn/cgi-bin/CRISPR2/CRISPR), E-CRISP (http://www.e-crisp.org/E-CRISP/index.html), and MultiTargeter (http://multicrispr.net/basic_input.html) to design sgRNAs. After that, the five sgRNAs of each gene with the highest scores were collected to construct the testing database. Comparing the sgRNA-design results of CIDP with the testing database, we found that most of sgRNAs designed by CIDP can be found in the testing database (87.4%, Fig. 1E). Moreover, the concordance of sgRNA-design results between CIDP and the other five tools were higher than 80% (Fig. 1E). These results indicated that the sgRNAs designed by CIDP remained highly reliable.

## Plans for the future

CIDP utilizes genomic sequences to build the database containing short segments whose sequences are unique across the whole genome. When designing sgRNAs, CIDP will extract candidate sgRNAs from gene sequences based on the selected PAM model, and then filter these sgRNAs by using the built database. Furthermore, CIDP will align the remaining sgRNAs to genome sequences to identify potential off-target sites and calculate off-target rates. Therefore, we believe that the sgRNAs designed by CIDP should be highly reliable, whereas, no matter the building or aligning process, the two operations were all performed at the level of genome. In general, a large amount of computer memory is required for supporting genome-level operations. This is an important reason why the tools performing genome-level operations can only be run on Linux servers [10, 11]. However, the memory of Windows or MacOS system is usually limited, which leads to low efficiency of CIDP.

In order to solve the efficiency problem of CIDP as much as we could, we developed a Linux version of CIDP that can be run on Linux servers. Moreover, the built database can be reused. Therefore, it is not required for users to build the database repeatedly. The CRISPR system is a common technology for wet experiments, and a species may be studied for several years by some wet labs. In this situation, the built database can also be reused by researchers in the same lab. It is truly hard to finish genome-level jobs under the condition of limited memory with high efficiency. Increasing CIDP efficiency is an important task for us in the future. In the next version, we will try to design functions in a multi-threaded or multi-processed manner which may elevate the design efficiency, even though these manners still require amount of memory support.

## Supplementary Material

Web_Material_uhad092

## Data Availability

CIDP written by python 3.8 is a stand-alone software that can be run in Windows, MacOS and Linux systems. The manual and tutorial videos as well as example data can be found in our github account (https://github.com/simon19891216/CIDP), and the CIDP software can be downloaded from https://github.com/simon19891216/CIDP/releases/tag/CIDPv1.2 or https://gitee.com/SimonX19891216/CIDP/releases/tag/CIDP.
