# Supplementary material for: CIDP: a multi-functional platform for designing CRISPR sgRNAs
Source: Hortic Res. 2023 May 4;10(7):uhad092. doi: 10.1093/hr/uhad092 (PMC10321375; doi:10.1093/hr/uhad092)
Supplement: Web_Material_uhad092 [file web_material_uhad092.docx]

**CIDP: A multi-functional platform for designing CRISPR sgRNAs**

Dong Xu^1#^, Jin Zhang^2#^, Xianjia Zhao^1#^, Yuze Hou^3^, Heling Jiang^1^, Wenchuang He^1*^, Xiongfeng Ma^4*^, Weihua Pan^1*^

^1^ Shenzhen Branch, Guangdong Laboratory for Lingnan Modern Agriculture, Genome Analysis Laboratory of the Ministry of Agriculture and Rural Affairs, Agricultural Genomics Institute at Shenzhen, Chinese Academy of Agricultural Sciences, Shenzhen, 518120, China

^2^State Key Laboratory of Subtropical Silviculture, College of Forestry and Biotechnology, Zhejiang A&F University, Hangzhou, Zhejiang 311300, China

^3^Taiyuan University of Technology, Taiyuan, Shanxi 030024, China

^4^State Key Laboratory of Cotton Biology, Institute of Cotton Research, Chinese Academy of Agricultural Sciences, Anyang 455000, China

# These authors contributed equally to this work.

*Correspondence: Wenchuang He, Email: hewenchuang@caas.cn; Xiongfeng Ma, Email: [maxf_caas@163.com](mailto:maxf_caas@163.com); Weihua Pan, Email: [panweihua@caas.cn](mailto:panweihua@caas.cn)

**Table S1 Characteristics of popular sgRNA design tools**

| **tool** | **require a reference** | **No. of plant models** | **Off-target score** | **Design common sgRNAs** | **Design in batch** |
| --- | --- | --- | --- | --- | --- |
| **CIDP** | **no** | **/** | **yes** | **yes** | **yes** |
| BE-Designer | yes | 142 | no | no | no |
| CCTop | yes | 49 | yes | no | no |
| CHOPCHOP | yes | 52 | yes | no | no |
| CRISPOR | yes | 139 | yes | no | no |
| MultiTargeter | yes | 3 | yes | no | no |
| CRISPRdirect | yes | 67 | yes | no | no |
| CRISPR-GE | yes | 35 | yes | no | no |
| CRISPR-P | yes | 65 | yes | no | no |
| E-CRISP | yes | 7 | yes | no | no |
| PlantPegDesigner | no | / | no | no | no |
| Sequence Scan for CRISPR | no | / | no | no | no |

Note: "/" means there was no limit on this term.
